# The “cardiac neglect”: a gentle reminder to radiologists interpreting contrast-enhanced abdominal MDCT

**DOI:** 10.3389/fcvm.2023.1147166

**Published:** 2023-04-25

**Authors:** Emina Talakić, Helmut Schöllnast, Ann-Katrin Kaufmann-Bühler, Florian Hohenberg, Ksenija Mijović, Eszter Nagy, Michael Fuchsjäger, Sebastian Tschauner

**Affiliations:** ^1^ Division of General Radiology, Department of Radiology, Medical University of Graz, Graz, Austria; ^2^ Institute of Radiology, LKH Graz II, Graz, Austria; ^3^ Department of Radiology, Charité Universitätsmedizin Berlin, Berlin, Germany; ^4^ Emergency Radiology Department, Center for Radiology and MRI, University Clinical Center of Serbia, Belgrade, Serbia; ^5^ Division of Paediatric Radiology, Department of Radiology, Medical University of Graz, Graz, Austria

**Keywords:** diagnostic imaging, multidetector computed tomography, contrast media, abdomen, abdominal pain, myocardial infarction, missed diagnosis

## Abstract

Myocardial infarction (MI) may be visible on contrast-enhanced multidetector computed tomography (MDCT) scans of the abdomen. In the previous literature, potentially missed MI in abdominal MDCTs was not perceived as an issue in radiology. This retrospective single-center study assessed the frequency of detectable myocardial hypoperfusion in contrast-enhanced abdominal MDCTs. We identified 107 patients between 2006 and 2022 who had abdominal MDCTs on the same day or the day before a catheter-proven or clinically evident diagnosis of MI. After reviewing the digital patient records and applying the exclusion criteria, we included 38 patients, with 19 showing areas of myocardial hypoperfusion. All MDCT studies were non ECG-gated. The delay between the MDCT examination and MI diagnosis was shorter in studies with myocardial hypoperfusion (7.4±6.5 hours and 13.8±12.5 hours) but not statistically significant p=0.054. Only 2 of 19 (11%) of these pathologies had been noted in the written radiology reports. The most common cardinal symptom was epigastric pain (50%), followed by polytrauma (21%). STEMI was significantly more common in cases of myocardial hypoperfusion p=0.009. Overall, 16 of 38 (42%) patients died because of acute MI. Based on extrapolations using local MDCT rates, we estimate several thousand radiologically missed MI cases worldwide per year.

## 1. Introduction

Myocardial infarction (MI) might present with atypical symptoms like epigastric pain and discomfort, or could be veiled by trauma after collapse due to nascent arrhythmias (1). Emergency physicians might misjudge the clinical symptoms, electrocardiograms can be normal despite MI, and laboratory parameters could be borderline or delayed (2). As a result, some patients undergo an emergency multidetector computed tomography (MDCT) of the abdomen because of suspected abdominal pathologies before the primary diagnosis is established (3).

Contrast-enhanced (CE) MDCT can visualize myocardial hypoperfusion (MH) in case of acute or chronic infarction (4). Radiologists might observe patchy alterations or defects in myocardial perfusion in the affected vascular territory (5) with a sensitivity of up to 94% in early phases and 75% in delayed phase (6). Especially in dedicated chest scans, protocols can be optimized to depict MH (7,8), or can even be detected in non-contrast enhanced dual-energy MDCT (9). Abdominal MDCTs cover the caudal parts of the heart frequently, then allowing to evaluate myocardial contrast attenuation and possibly suspecting ischemia, mostly covered in terms of case reports (4,10–12).

Our motivation for this retrospective data analysis was that we occasionally observed incorrectly triaged patients who received emergency abdominal MDCT in the setting of MI, visible but unrecognized by the radiologist. We wanted to assess whether and to what extent this could be a clinically relevant problem.

## 2. Materials and methods

We queried the local hospital information system (HIS) of the University Hospital Graz, Austria for patients who underwent CE abdominal MDCTs the same day or the day before being ICD-10 (International Classification of Diseases 10th Revision) coded with “I21.*,” resembling “acute myocardial infarction.” 107 patients met the stated search criteria between 2006 and 2022.

**Figure 1:**
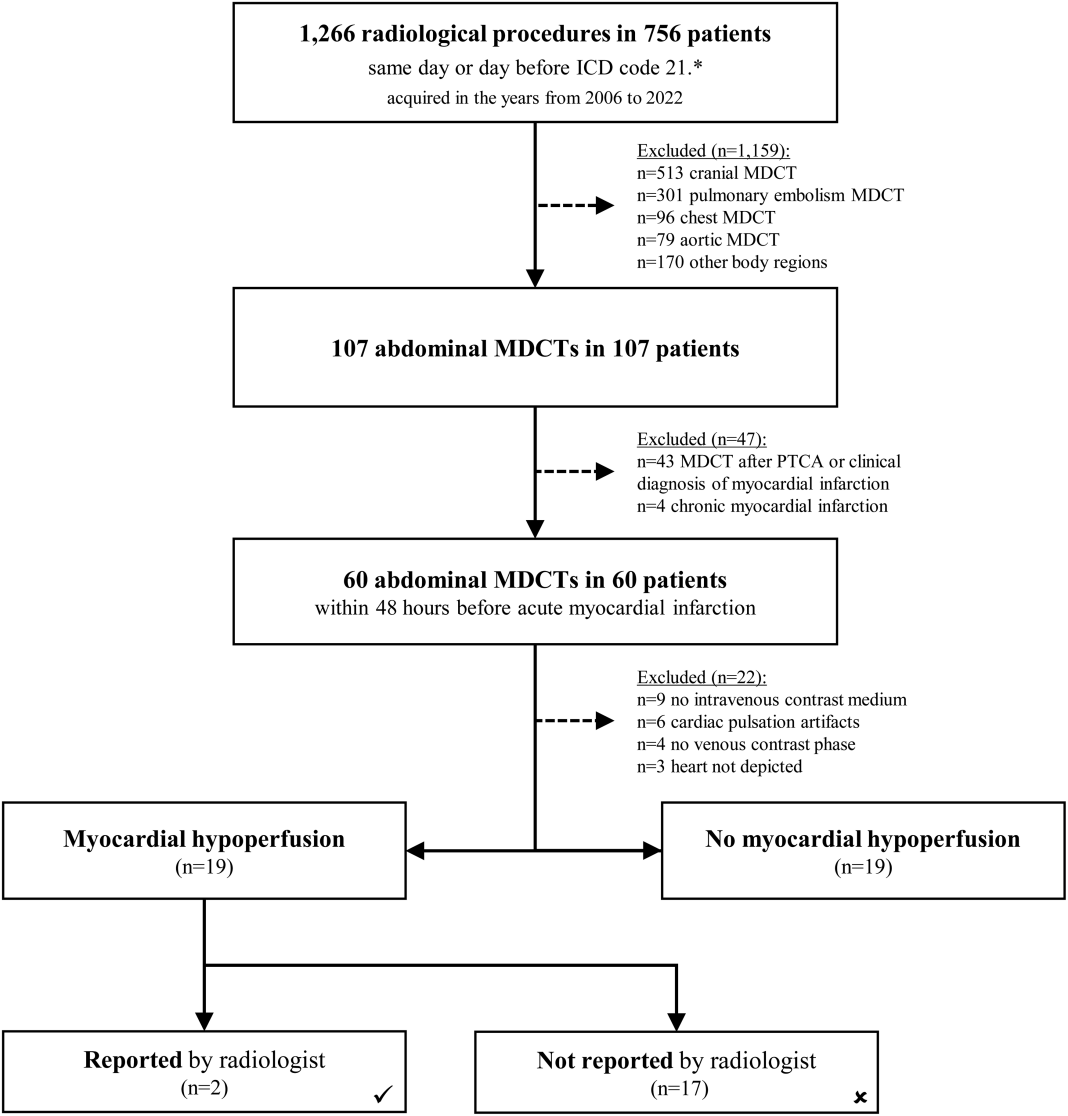
Flow-chart of all patients assessed for myocardial hypoperfusion. All eligible MDCT examinations were screened for the presence of myocardial hypoperfusion.

The local HIS was queried for the dates and times of percutaneous transluminal coronary angioplasties (PTCA) or the clinical diagnosis of acute MI, subsequently cross-referenced with the MDCT examinations to calculate the time difference. The timestamps were manually checked for accuracy and corrected if necessary. This procedure lead to the exclusion of 38 studies which had been performed after the diagnosis of acute MI (compare Figure 1). In addition, we researched the leading clinical symptoms and searched the radiological report texts for descriptions of MH. We also noted the type of MI (non-ST elevation myocardial infarction = NSTEMI and ST elevation myocardial infarction = STEMI) and relevant abdominal pathologies visible in the studies. Another parameter of interest was the clinical outcome in terms of death or discharge. We retrieved the cardiac Troponin T (cTn) or, when available, the high-sensitivity cardiac Troponin T (hs-cTn) values in micrograms per liter (μg/l) or nanograms per liter (ng/l) respectively, as laboratory indicators of MI, including their timestamps.

Radiforce RX340 10-bit color monitors (EIZO Corporation, Hakusan, Ishikawa, Japan) displayed the examinations retrieved by a syngo.plaza workstation version VB30C_HF06 (Siemens Healthineers, Erlangen Germany). Three Radiologists (E.T., K.M., S.T.) consensually reviewed the MDCT examinations for interpretability and any myocardial perfusion deficits. The raters classified studies as inadequate when lacking CE, missing venous CE phase, incorrect chronological order, missing depiction of the heart, or pulsation artifacts.

**Figure 2:**
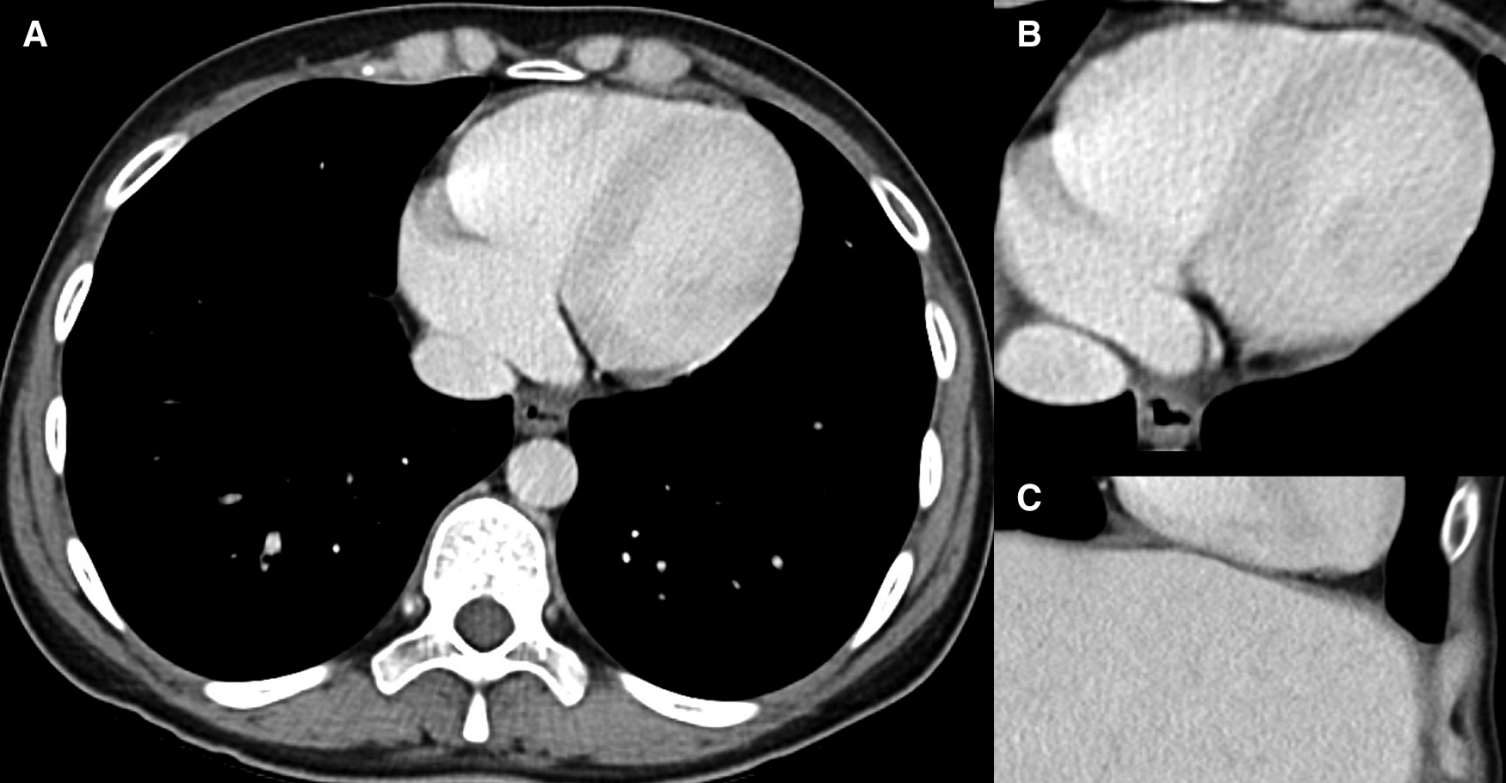
Normal cardiac contrast enhancement. 40-year-old male patient who underwent CE MDCT of the abdomen in (**A**,**B**) axial orientation and (**C**) coronal section. Note the homogeneous contrast of the myocardium, as far as covered.

Figure 2 showing an example of a heart on CE abdominal MDCT with normal and homogeneous contrast enhancement. Note that due to the usually thicker left ventricular myocardium, contrast enhancement is often easily detectable left ventricularly and septally, whereas perceptibility may be limited in the right ventricle.

Statistical tests were performed with SPSS v21 (SPSS Inc., Chicago, IL, USA). We calculated descriptive statistics and, in the case of an assured normal distribution, using independent samples t-tests. Chi-square test was used for nonparametric data. P values less than 0.05 were considered statistically significant. The study population contained a total of 38 patients who remained after applying the exclusion criteria (compare flowchart Figure 1). Two subgroups were statistically compared, with and without obvious MH (each n=19 per group). Hypotheses tested included that STEMI would be seen more frequently in the MH group, the delay between MDCT and MI diagnosis would be shorter in the MH group, and that patients in the MH group would be younger.

## 3. Results

We identified 756 patients who have undergone a total of 1,266 MDCT procedures (body regions) the same day or the day before the diagnosis of MI was established. 107 of these patients were referred to abdominal MDCT. Further scan regions were brain (n=513), chest (n=96, pulmonary embolism n=301), aorta (n=79), and various other body regions (n=170) (Figure 1). The mean age of these patients was 69.2±14.3 years (range 32 to 97 years). 63 (58.9%) were male and 44 (41.1%) female patients. 47 patients were excluded in the next step due to MDCTs after PTCA or clinical diagnosis of MI (n=43), or chronic MI (n=4). Further 22 examinations were excluded because of missing contrast attenuation, cardiac pulsation artifacts, only arterial enhancement, or missing depiction of the heart. None of the studies was ECG-gated. We included 38 studies suitable for analysis of MH (Figure 1). In 19 of 38 cases (50%) MH was evident, while in 19 cases there were no hints of MH. Radiologists had documented only 2 of 19 (11%) MHs in their written reports.

STEMI was significantly more common in the group of patients with MH, with 14 of 19 cases vs. 6 of 19 cases, Chi-shqare test p=0.009. 14 of 19 MH patients (74%) underwent PTCA after MDCT, while the rest were diagnosed clinically alone. The delay between the MDCT examination and the MI diagnosis was shorter in the group of studies with MH [7.4±6.5 hours (range 1.1 to 20.7 hours) vs. 13.8±12.5 hours (range 1.1 to 47.2 hours)] but not statistically significant (t-test p=0.054). Patients in the MH group were younger on average (65.4±14.3 years vs. 72.1±11.5 years, t-test p=0.122), though not statistically significant.

**Figure 3:**
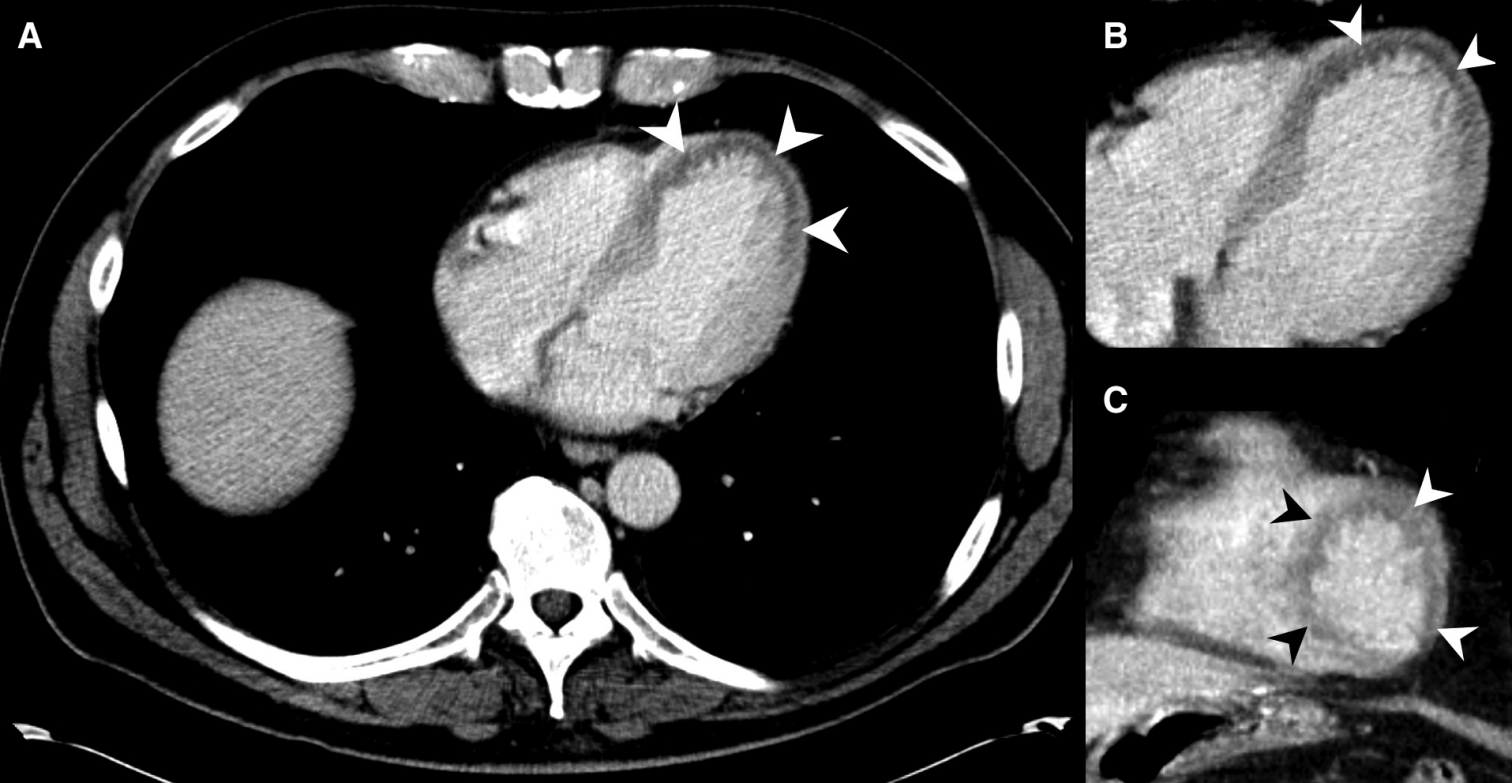
MDCT of a 53-year-old male presenting pain in the chest and upper abdominal area. MH is noted at the left ventricle at the heart apex on axial slices (**A**,**B**) and on coronal reformations (**C**) (arrowheads), but was not described in the radiological report. Emergency doctors clinically suspected MI about one hour after the MDCT, and the patient was transferred to PTCA shortly after. The patient could be discharged from hospital after a few more days.

Death as main parameter of outcome was not significantly different between patients showing MH on CE MDCT (9 of 19=47.4%) compared to patient not demonstrating MH (7 of 19=37%), Chi-shqare test p=0.511. In total, 16 of 38 (42%) patients died as a consequence of acute MI, while the rest was discharged from the hospital in stable condition (compare case in Figure 3).

The cardinal symptoms of patients who underwent MDCT within 48 yours before the diagnosis of MI (n=38) were:

• Abdominal pain (n=19) 50.0%
• Polytrauma (n=8) 21.1%
• Chest pain (n=2) 5.3%
• Dyspnea (n=3) 7.9%
• Cardiac arrest (n=3) 7.9%
• Syncope (n=3) 7.9%

Cardiac Troponin (cTn and hs-cTn) values were not taken earlier or later in patients with MI (n=19, mean 8.3±19.2 hours after the MDCT, median 0.7 hours, range −12.5 to 79.2 hours) and without MI (n=19, mean 6.9±11.2 hours after the MDCT, median 4.1 hours, range −1.9 to 43.9 hours), Chi-shqare test p=0.264. 15 out of 19 (79%) patients demonstrating MH had elevated levels of cTn or hs-cTn, as can be noted in Table 1 by a plus (+) symbol.

**Table 1:** Sex, age, cardinal symptoms, MI type, delay between MDCT and diagnosis, radiologist reports, cardiac Troponin T values and clinical outcome of patients with MH.

Sex, Age (years)	MI type	Cardinal symptom	Delay between MDCT and diagnosis (hours)	MH reported by radiologist	Cardiac Troponin values	Outcome
m, 72	NSTEMI	Abdominal pain	8.5	no	hs-cTn 988 ng/l +	discharged
m, 76	NSTEMI	Abdominal pain	3.3	no	hs-cTn 14,927 ng/l +	discharged
f, 68	STEMI	Abdominal pain	3.2	no	cTn 0.15 μg/l	discharged
m, 79	STEMI	Abdominal pain	18.7	no	cTn 1.63 μg/l +	discharged
m, 53	STEMI	Abdominal pain	9.0	yes	hs-cTn 1,563 ng/l +	discharged
f, 81	STEMI	Abdominal pain	16.2	no	cTn 2.77 μg/l +	deceased
f, 83	NSTEMI	Abdominal pain	11.3	no	hs-cTn 2,661 ng/l +	deceased
m, 73	STEMI	Abdominal pain	5.8	no	hs-cTn 1,659 ng/l +	deceased
m, 43	STEMI	Cardiac arrest	5.6	yes	hs-cTn 67 ng/l +	deceased
m, 53	STEMI	Chest pain	2.1	no	cTn 0.68 μg/l +	discharged
m, 65	NSTEMI	Dyspnea	2.1	no	cTn 0.32 μg/l	discharged
f, 78	NSTEMI	Dyspnea	4.4	no	hs-cTn 5,165 ng/l +	deceased
m, 67	STEMI	Dyspnea	20.7	no	cTn 0.09 μg/l	deceased
m, 73	STEMI	Polytrauma	18.3	no	cTn 0.02 μg/l	deceased
m, 48	STEMI	Polytrauma	3.3	no	hs-cTn 388 ng/l +	discharged
f, 44	STEMI	Polytrauma	1.1	no	hs-cTn 858 ng/l +	discharged
m, 55	STEMI	Polytrauma	2.7	no	hs-cTn 1,712 ng/l +	discharged
m, 45	STEMI	Polytrauma	2.9	no	hs-cTn 72 ng/l +	deceased
m, 86	STEMI	Polytrauma	1.8	no	hs-cTn 44 ng/l +	deceased

The examiners assessed the abdominal MDCT slices for signs of clinically relevant acute abdominal pathologies which could explain a patient’s symptoms. Only one patient in the MH group featured signs of acute abdominal pathology, namely cholecystitis (Table 1). The majority of the examinations did not demonstrate such hints:

• No acute abdominal pathology (n=31) 81.6%
• Liver laceration (n=1) 2.6%
• Bowel ischemia (n=2) 5.3%
• Abdominal abscess (n=1) 2.6%
• Diverticulitis (n=1) 2.6%
• Splenic laceration (n=1) 2.6%
• Cholecystitis (n=1) 2.6%

## 4. Discussion

Our retrospective data evaluation of MH in CE MDCTs of the abdomen starting in 2006 showed a substantial rate of unreported cases of MI. Despite a long-standing knowledge of the potential detectability of MH, to date no studies estimated the clinical relevance of this topic and researchers have not yet perceived the issue of potentially missed MH. Radiologists could help shorten delays to MI diagnosis or PTCA in some cases by taking a quick look at myocardial contrast attenuation.

**Figure 4:**
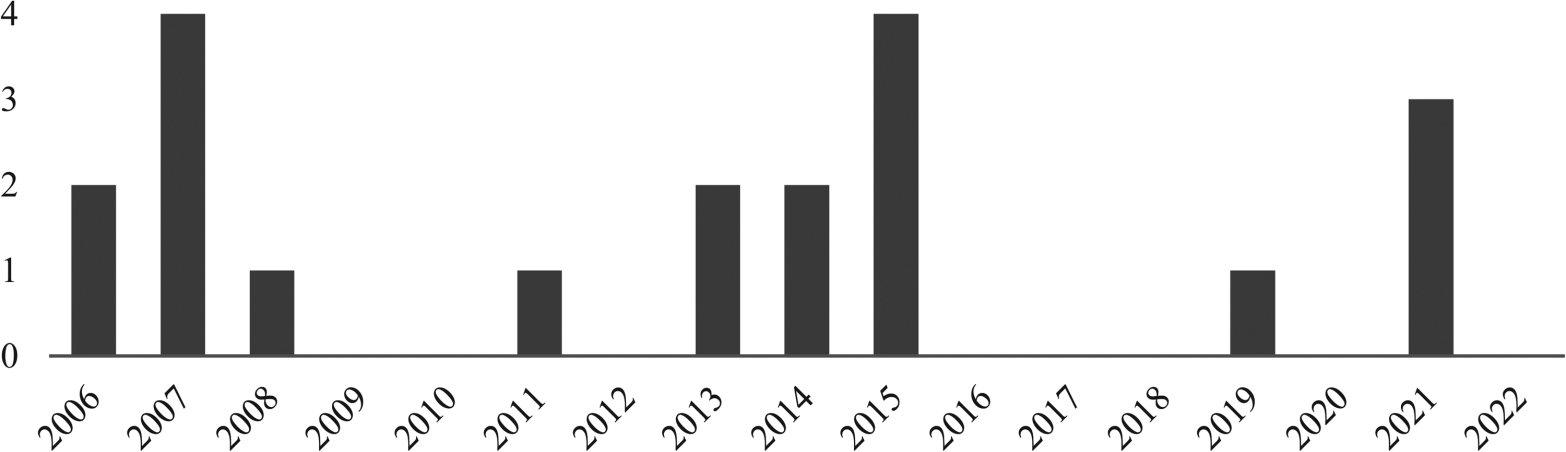
Chart of detect MH cases per year from 2006 to 2022 at the authors’ institution. A yearly rate of 0 to 4 patients with MH was noted.

**Figure 5:**
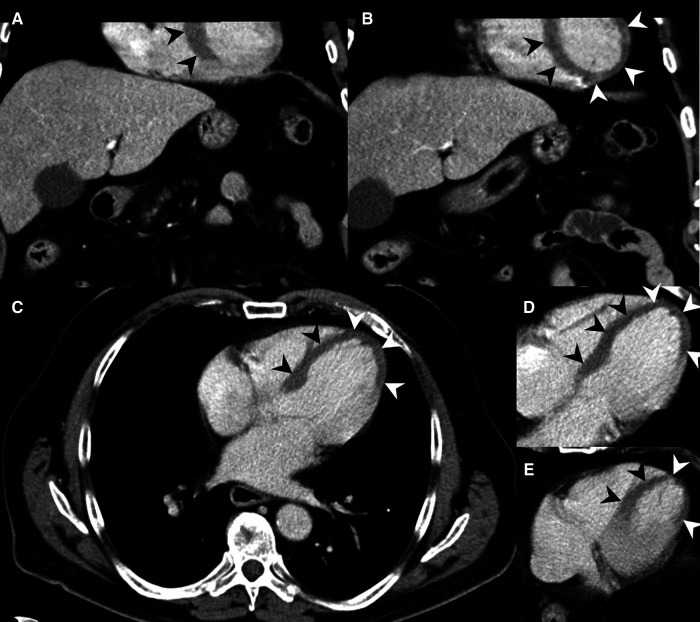
73-year-old male with acute MI, evident as MH on MDCT (arrowheads). (**A**,**B**) Coronal reformations show large areas of MH in the left ventricle. (**C**–**E**) MH shown at the interventricular septum and the heart apex in axial slices. The patient deceased after PTCA.

**Figure 6:**
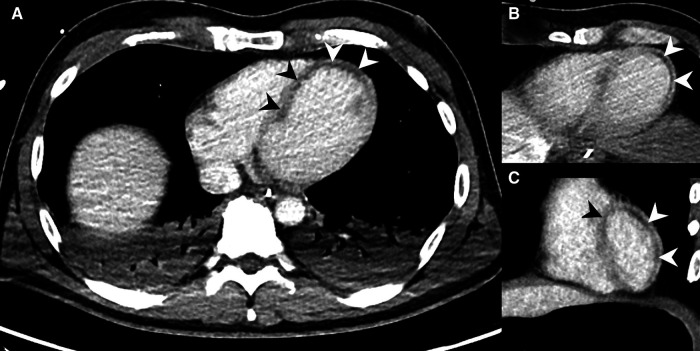
MDCT examination of a 43-year-old male, who was required to be resuscitated in the emergency room with suspected pulmonary embolism. The patient was referred for MDCT with a delay of three hours. The abdominal MDCT series showed MH (arrowheads) in the vascular territory of the left anterior descending artery (LAD) in axial slices (**A**,**B**) and coronal reformations (**C**), which was immediately communicated to the treating physicians. Stents were placed in both coronary arteries during emergency PTCA. The patient died two days later as a result of his MI.

Our institution performed 35,671 MDCT examinations in 2021, 5,743 of it covering the abdomen. We detected 19 cases of MH on abdominal CE MDCT in our hospital over the last 15 years, resulting in a rate of at least 1.2 cases per year (Figure 4). It is likely that this number is higher, as limitations in filtering appropriate cases and the common delays in coding ICD-10 data in daily practice suggest a sizeable number of undetectable cases. Extrapolated to the estimated 300 million CT scans per year (13) and assuming a comparable number of missed diagnoses as in our hospitals, there might be a few thousand related MH cases not reported by radiologists around the globe each year. This appears even more alarming when reflecting the high mortality (47%) in our patient population with visible MH (compare cases in Figure 5, 6).

**Figure 7:**
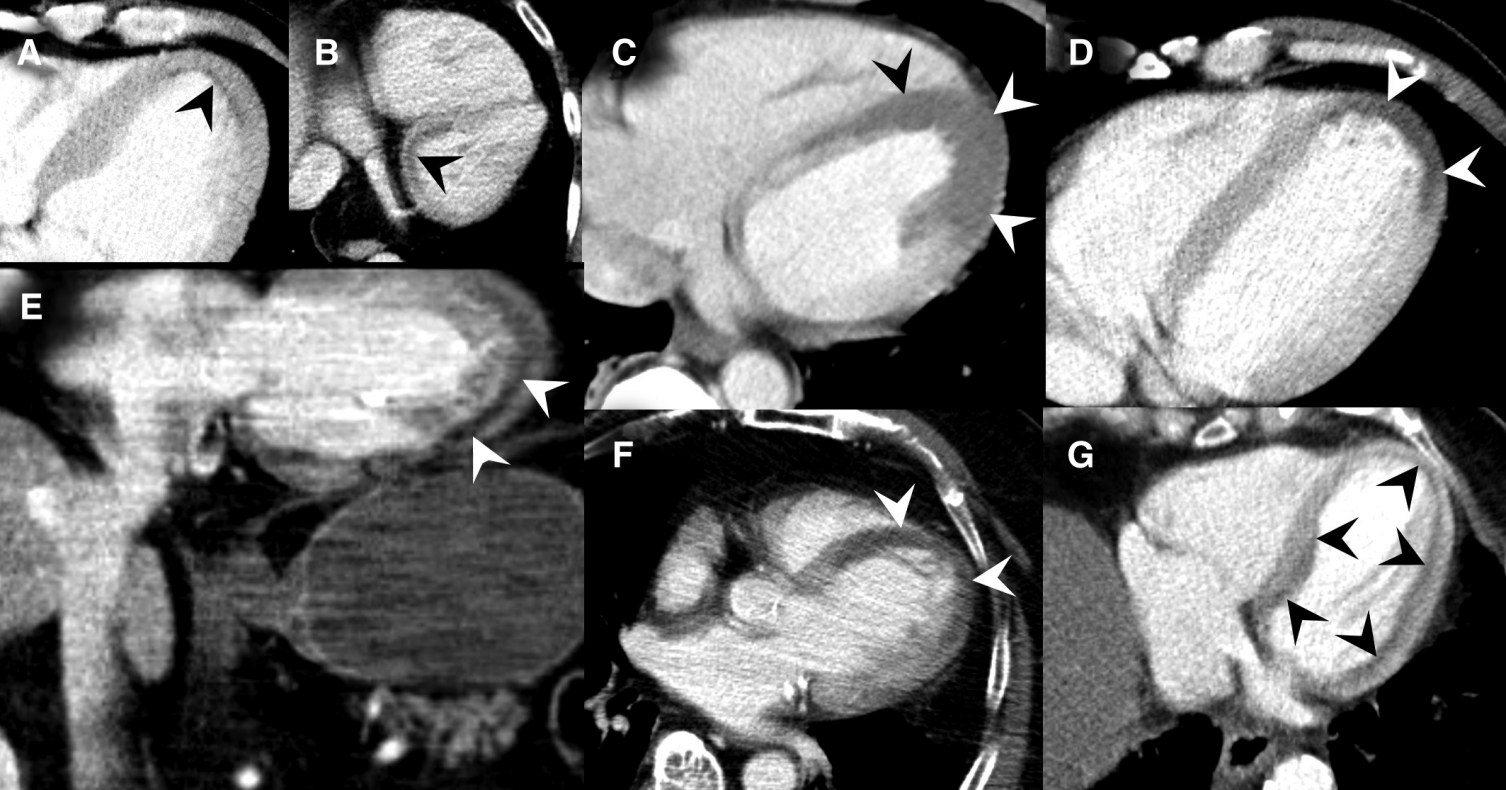
Collection of MDCT images of patients with MH (black and white arrowheads) caused by acute MI. (**A**) Slight subendocardial perfusion deficit at the heart apex. (**B**) Coronal slice through the heart base showing predominately subendocardial MH. (**C**) Extensive MH at the left ventricle. (**D**) Coronal reformats with impressive MH at the cardiac apex. (**E**) Axial MDCT slices demonstrating MH at the cardiac apex. (**F**) Subendocardial MH along the entire left ventricle.

MH is often not difficult to detect (4), as depicted in Figure 7. The problem seems that radiologists do not regularly think about the eventuality of MH. Referrals like abdominal pain, polytrauma, and dyspnea were the most common clinical symptoms in patients with MH. Radiologists looking at the cardiac contrast attenuation closely could reduce PTCA treatment delays, which were 7.1 hours in our missed cases on average.

Our analyses of the report texts and the available imaging material revealed few indications that the clinical complaints did not point to MI as the primary cause. Radiologists should be especially alert if the referral diagnosis includes epigastric pain or upper abdominal pain, if respiratory distress or resuscitation is being described, or if polytrauma is present. All patients we identified, had some sort of acute symptoms. In the group of patients with MH, one gallbladder wall thickening was described (Table 1) as possible acute abdominal pathology. This wall thickening was interpreted as cholecystitis, but might as well be caused by edema due to heart insufficiency. It is extremely unlikely that MH will be seen as true ”incidental” finding in patients undergoing a routine MDCT examinations like in the setting of cancer staging.

A noteworthy observation during our evaluation was the fact that patients were most frequently referred for MDCT of the brain shortly before the diagnosis of acute MI (Figure 1). This might be caused by patients suffering from cranial hypoperfusion demonstrating neurological symptoms and syncopes. Also scans for pulmonary embolism were frequently performed, underlining the difficulties in clinically discriminating different sources of chest pathologies.

Radiological visualization of MH does typically not reveal the onset, apart from cases where calcifications or fatty atrophy of the myocardium are present. Among the excluded cases, we detected another 13 patients who demonstrated MH either as chronic condition or post PTCA treatment. Medical history records could help to decide whether a chronic MI situation is present or not.

The limitations of our data analysis are primarily attributable to the retrospective study design. One of the main issues is the low number of appropriate patients, whereby we have to assume a high number of unidentified cases due to the limited search and filtering possibilities within our IT systems. Because only two MH cases were included in the corresponding written reports, we could not calculate, whether these patients could gain a statistical advantage from earlier diagnoses. Larger studies are warranted in the future to enable more insights into this important topic.

## 5. Conclusions

Myocardial hypoperfusion may be visible on contrast-enhanced emergency MDCT of the abdomen in the context of myocardial infarction, but is regularly missed by radiologists. Thus, radiologists are advised to think about the possibility of myocardial infarction in cases of upper abdominal pain, polytrauma, chest pain, or dyspnea, especially when no signs of abdominal pathology are visible.

## Data Availability

The raw data supporting the conclusions of this article will be made available by the authors, without undue reservation.
